# Pre-Competition Stress in Female Volleyball Players: The Role of Experience, Sleep, and Coping

**DOI:** 10.3390/healthcare14020155

**Published:** 2026-01-07

**Authors:** Kamila Litwic-Kaminska

**Affiliations:** Faculty of Psychology, Kazimierz Wielki University, Staffa 1, 85-867 Bydgoszcz, Poland; k.litwic@ukw.edu.pl

**Keywords:** stress, sleep quality, female athletes, coping strategies, volleyball

## Abstract

**Highlights:**

**What are the main findings?**

- About 60% of female volleyball players reported poor sleep quality before competition, regardless of league level.
- Athletic experience, sleep quality, and reliance on social support predicted perceived stress, with less experienced athletes showing higher stress levels.

**What are the implications of the main findings?**

- Developing adaptive coping strategies and improving sleep hygiene may reduce pre-competition stress among female athletes.
- Targeted psychological and recovery programs are particularly important for less experienced players preparing for competition.

**Abstract:**

**Background/Objectives**: Athletes face both daily and sport-related stressors while being expected to perform at an optimal level. Effective recovery, particularly adequate sleep, plays a key role in psychophysiological restoration and performance, whereas sleep deprivation may impair functioning and increase perceived stress. This study examined the associations between coping strategies, sleep quality, athletic experience, competitive level, and perceived stress during the pre-competition period among female volleyball players. **Methods**: Ninety-one athletes (aged 18–35, M = 23.03, SD = 4.37) from three Polish professional leagues—Tauron (*n* = 31), First League (*n* = 30), and Second League (*n* = 30)—completed an online battery including the Stress Coping Strategies in Sport Questionnaire (SR3S), the Perceived Stress Scale (PSS-4), the Pittsburgh Sleep Quality Index (PSQI), and a demographic survey. **Results**: Based on PSQI scores, approximately 60% of the athletes were classified as poor sleepers. No significant differences in sleep quality or perceived stress were found across leagues. However, athletes competing in higher leagues reported more frequent use of mental coping strategies. Athletic experience, sleep quality, and the coping strategy of seeking social support were significantly associated with perceived stress. Players with less experience, poorer sleep, and a greater tendency to seek social support reported higher stress levels. The positive association between support-seeking and stress likely reflects reactive coping among more stressed athletes rather than a maladaptive effect of social support. **Conclusions**: These findings underscore the importance of promoting adaptive coping and sleep hygiene in competitive sport, particularly among less experienced female athletes during the pre-competition period.

## 1. Introduction

Competitive volleyball demands exceptional physical fitness, technical proficiency, and psychological resilience. At elite levels, performance margins are minimal, increasing the importance of mental preparation [1,2]. Athletes are exposed to a wide range of stressors that intensify in the days preceding competition, including evaluative pressure, uncertainty about outcomes, travel-related fatigue, and role-related demands. Pre-competition stress, defined as heightened physiological arousal and psychological tension before an athletic event, represents a multidimensional phenomenon affecting athletes across all performance levels [1].

In volleyball, where split-second decisions and precisely coordinated team actions are required, the ability to regulate pre-competition anxiety may be important for performance quality [2]. Female volleyball players face not only individual performance expectations, but also complex interpersonal demands related to communication, tactical coordination, and role clarity within the team. These social and organizational factors may further intensify stress responses and shape preferred coping strategies [3,4]. Temporal analyses of competitive anxiety indicate that both cognitive and somatic components of anxiety tend to increase as competition approaches, while self-confidence often decreases in the hours preceding a match [2]. This pattern appears particularly pronounced among female volleyball players, for whom team dynamics and role-specific expectations can additionally amplify stress reactions [1].

Previous research has shown that female athletes often report higher levels of cognitive anxiety and worry-related symptoms than their male counterparts in competitive settings [1,5]. Gender-related differences have also been observed in coping preferences, with women more frequently relying on emotion-focused strategies, whereas men tend to use problem-focused approaches [6,7]. In team sports such as volleyball, however, coping is not solely an individual process but is embedded within collective functioning. Athletes who are able to integrate personal coping strategies with team-based support systems tend to show better emotional regulation and more stable performance [8,9].

The effectiveness of coping strategies among female volleyball players varies substantially. Adaptive strategies, including cognitive reframing, structured behavioural routines, and emotional regulation, are associated with more favourable psychological outcomes and reduced negative effects of competitive anxiety [10,11]. In contrast, avoidance-oriented coping and inconsistent stress management are linked with greater vulnerability to pre-competition stress [6,8]. Importantly, effective coping is not limited to reducing anxiety intensity but also involves functional interpretation and the utilization of arousal states [10,12]. From a transactional perspective, coping is therefore a dynamic process shaped by continuous interactions between individual characteristics and situational demands [13].

Sleep quality is another important factor associated with pre-competition functioning. Both narrative and systematic reviews consistently report that a substantial proportion of athletes experience insufficient or poor-quality sleep, particularly in the period surrounding competitions [14,15,16,17]. Sleep disturbances have been associated with impairments in vigilance, decision-making, emotional regulation, and physical performance [18,19]. Anticipatory stress, altered routines, and travel demands commonly contribute to sleep deterioration before competition, reflecting the bidirectional nature of the stress–sleep relationship. Pre-competition anxiety may disrupt sleep, while sleep restriction is associated with higher perceived stress, creating a self-reinforcing cycle [20,21]. Female athletes appear to be especially vulnerable to this pattern, with worry and perceived stress identified as important correlates of poor sleep [5,22]. Evidence also suggests that sleep obtained 48–72 h before competition may be particularly relevant for subsequent performance outcomes [14,23]. Psychological resources and sport experience further modify the stress–sleep–coping relationship. For example, mental resilience has been shown to buffer the association between perceived stress and sleep quality among female athletes [24]. Progressive exposure to sport-specific demands may foster the development of more effective coping strategies over time. Athletic experience plays a significant role in shaping pre-competition responses [25]. More experienced athletes typically report a broader repertoire of coping strategies and more stable pre-competition routines [26,27]. They are also more likely to appraise stressful situations as challenges rather than threats, which supports confidence stability and behavioural flexibility [11,28]. At the same time, experience does not uniformly protect against stress, as experienced players may face additional pressures related to performance expectations, leadership roles, and career maintenance [6,8]. Less experienced athletes, in turn, often report higher levels of pre-competition anxiety and less consolidated coping routines, which may increase susceptibility to stress-related sleep disturbances [24,29].

Taken together, prior findings support a multilevel, transactional framework in which (a) sleep quality tends to deteriorate as competition approaches, (b) coping strategies—particularly cognitive and well-rehearsed routines—are closely linked with both stress and sleep, and (c) gender and athletic experience function as important contextual moderators [1,7,17,29]. Despite the breadth of research on pre-competition anxiety, relatively few studies have simultaneously examined the combined roles of sleep quality, coping strategies, and athletic experience, especially within samples of female team-sport athletes [7,24,29]. Drawing on the theoretical model of interactive stress regulation [13], it is assumed that an athlete’s psychophysical state, coping strategies, individual dispositions, and sport-specific contextual factors jointly shape stress appraisal and stress responses, particularly during periods of heightened pre-competition tension. Accordingly, the present study aimed to examine whether athletic experience, sleep quality, and coping strategies are associated with perceived stress during the pre-competition period among female volleyball players competing across three professional levels. In formulating the research framework, the following hypotheses were proposed:

**H1:**  *Poorer sleep quality is associated with higher levels of perceived pre-competition stress.*

**H2:**  *Greater athletic experience is associated with lower levels of perceived pre-competition stress.*

**H3:**  *The use of problem-focused coping strategies (e.g., planning/focus on activity or applying mental techniques) is associated with lower perceived stress, whereas greater reliance on support-seeking is associated with higher perceived stress.*

**H4:**  *Competitive level differentiates the level of perceived stress and sleep quality as well as use of selected coping strategies.*

## 2. Materials and Methods

### 2.1. Study Design

The study employed a cross-sectional correlational design. Participants were recruited from volleyball clubs across Poland using purposive sampling. Eligibility criteria included active membership in a professional volleyball league, regular participation in training sessions, and absence of injury preventing match participation immediately following the assessment. Participation was voluntary, and athletes were invited individually to take part in the study.

Data were collected three days before a scheduled match using an anonymous online survey (Google Forms). All participants completed the questionnaires on the same relative day within their competition cycle. Informed consent was obtained through submission of the form. The study protocol adhered to the ethical standards outlined in the Declaration of Helsinki and was approved by the Ethics Committee for Scientific Research at the Faculty of Psychology, Kazimierz Wielki University (Poland). Participants were informed about the purpose and scope of the study, and confidentiality of responses was assured. Questionnaires were completed individually, without the presence of coaches or teammates. The researcher was available to provide clarification if needed.

### 2.2. Participants

The sample comprised 91 female volleyball players aged 18–35 years (M = 23.03, SD = 4.37) representing three professional leagues in Poland: Tauron League (*n* = 31), First League (*n* = 30), and Second League (*n* = 30). Athletic experience ranged from 3 to 25 years (M = 11.82, SD = 4.52). Eight First League and seven Second League players reported prior experience in a higher league. Detailed participant characteristics are presented in Table 1.

### 2.3. Methods

Participants completed an online battery of questionnaires assessing sleep quality, coping strategies in sport, perceived stress, and demographics (age, athletic experience, league history):

Pittsburgh Sleep Quality Index (PSQI, [30]). This 19-item instrument assesses subjective sleep quality and sleep disturbances over the past month. It comprises four descriptive items (bedtime, wake time, sleep duration, and sleep latency) and fifteen items rated on a 4-point scale (0–3). Responses are combined into seven components that sum to a global score ranging from 0 to 21, with higher scores indicating poorer sleep quality. Scores > 5 classify individuals as poor sleepers. The original Cronbach’s α was 0.83, indicating good internal consistency [30]. In the present study, the PSQI was used to capture habitual sleep quality during the weeks leading up to competition, rather than sleep obtained immediately before a match, as accumulated sleep patterns are considered relevant for stress regulation and pre-competition readiness [14,17,23].

Sport Stress-Coping Strategies Questionnaire (SR3S; [31]). The SR3S consists of 22 items about possible actions and reactions undertaken in stressful situations, rated on a five-point scale, grouped into four subscales: setting the goal/victory, seeking support, applying mental techniques (e.g., relaxation), and planning/focusing on the activity. Reported internal consistency ranges from 0.75 to 0.83 [31], with alphas of 0.82–0.89 obtained in the present study.

Perceived Stress Scale, *PSS-4* [32]. The four-item short form of the PSS-10 measures the degree to which individuals perceive situations in their lives as stressful during the past month. Items are rated on a five-point Likert scale, producing total scores from 0 to 16, with higher scores reflecting greater stress levels. In this study, the PSS-4 was used to assess general perceived stress as a background cognitive–emotional load present during the pre-competition period, rather than momentary competitive anxiety. The PSS-4 demonstrates satisfactory reliability (Cronbach’s α = 0.71) and psychometric properties comparable to the PSS-10 and PSS-14 [33,34].

### 2.4. Statistical Analyses

Analyses were conducted using Statistica v.13.3 (StatSoft, Tulsa, OK, USA). The normality of the distribution of variables was assessed using the Shapiro–Wilk test, along with evaluations of skewness and kurtosis. Differences between leagues were examined using the one-way ANOVA. The magnitude of the effect was indicated by the partial eta squared. Pearson R correlation was applied to determine the relationship between all continuous variables. A stepwise regression analysis was performed with coping strategies, sleep quality, league level, and sporting experience as independent variables. The perceived stress served as the dependent variable. A sensitivity power analysis indicated that with a sample size of N = 91 and α = 0.05, the study had adequate statistical power (1 − β ≥ 0.80) to detect at least medium-sized effects in one-way ANOVA (η^2^p ≈ 0.06) as well as medium effect sizes in multiple regression analyses (f^2^ ≈ 0.15).

## 3. Results

### 3.1. Stress, Coping, Sleep Quality, and Volleyball League

Descriptive statistics for female volleyball players across leagues are presented in Table 1. Second-league players reported the highest mean stress levels; however, no significant between-league differences were found (F (2, 88) = 0.86, *p* = 0.425, η^2^p = 0.02).

Across all groups, the goal/victory-oriented coping strategy yielded the highest scores. In contrast, the use of mental techniques was generally rated lowest. Tauron League players reported the lowest values for support-seeking. A significant league effect emerged only for the use of mental techniques (F (2, 88) = 4.15, *p* = 0.019, η^2^p = 0.09), with higher-league athletes reporting more frequent use.

The mean PSQI global score indicated reduced sleep quality (M = 5.59, SD = 2.53), with no significant differences between leagues (F (2, 88) = 2.44, *p* = 0.093, η^2^p = 0.05). Daytime dysfunction was the most common complaint, while the use of sleep medication was least frequent. Based on the PSQI cut-off (>5), approximately 60% of athletes were classified as poor sleepers, with similar proportions observed across the Tauron League (58%), First League (60%), and Second League (63%).

### 3.2. Partial Correlations

The correlation matrix showed that greater sport experience was weakly associated with lower perceived stress and better sleep quality. Higher perceived stress was moderately related to poorer sleep quality. Coping strategies showed low to moderate intercorrelations, with the strongest associations observed between planning/focus on activity and other task-oriented strategies. No evidence of excessively high correlations was found, indicating an absence of multicollinearity among the variables (Table 2).

### 3.3. Significant Predictors of Stress Levels in Female Volleyball Players

To identify predictors of perceived stress and the proportion of variance explained, a stepwise regression analysis was performed (Table 3). Regression model explained 20% of the variance in stress levels (F (5,85) = 4.30; *p* < 0.002). Significant predictors of stress were sports experience (β = −0.25; t = −2.40; *p* = 0.019), overall sleep quality (β = 0.25; t = 2.50; *p* = 0.015), and a coping strategy focused on seeking support (β = 0.23; t = 2.21; *p* = 0.030).

## 4. Discussion

Drawing on the interactive model of stress [13], this study assumes that athletes’ stress experiences emerge from the interplay of individual, situational, and discipline-specific factors rather than from any single determinant. This perspective is particularly useful for interpreting the observed pattern of results, including the absence of league-level differences in perceived stress.

Unexpectedly, perceived stress did not differ significantly across league levels. This finding is consistent with previous evidence showing that competitive level alone tends to be a weak predictor of stress intensity in team sports. Stress responses appear to be shaped by contextual and personal factors rather than by performance level per se [6,7,35]. A plausible explanation is a compensatory mechanism whereby higher competitive demands in top leagues are offset by more developed psychological skills, greater competitive experience, and more structured pre-competition routines [36,37]. In contrast, lower-league athletes may experience fewer sport-specific pressures but face stressors related to role instability, limited organizational resources, and balancing sport with non-sport obligations [6,7]. Despite qualitative differences in stressors, these demands may result in comparable levels of perceived stress [27,35]. Additionally, shared psychosocial conditions across female volleyball teams, such as similar team dynamics and competition schedules, may further contribute to comparable levels of perceived stress across leagues [38,39].

This interpretation is supported by the finding that higher-league athletes reported more frequent use of mental techniques for coping with stress [37]. Mental skills training, including imagery, self-talk, relaxation techniques, and mindfulness, is increasingly recognized as an integral part of preparation for competitive sport [36]. Athletes competing at higher levels typically have greater access to psychological support and are embedded within organizational cultures that prioritize psychological skills development [40]. These techniques may not eliminate stress but instead enhance athletes’ capacity to regulate it effectively. In this sense, advanced coping skills may buffer the impact of heightened competitive demands [35,36,40]. This may help prevent stress escalation, resulting in stress levels comparable to those observed in lower leagues [36,41,42]. However, given the cross-sectional design, causal conclusions regarding the development of these skills cannot be drawn. Longitudinal research is necessary to investigate how coping competencies develop throughout athletes’ careers.

Approximately 60% of athletes in the present study were classified as poor sleepers, with similar prevalence across leagues. This finding aligns with prior research demonstrating a high rate of sleep disturbances among athletes in the pre-competition period [17,18,22,23,29]. Female athletes may be particularly vulnerable to sleep disruption due to heightened emotional reactivity, increased sensitivity to interpersonal stressors, and greater cognitive preoccupation before competition [5,20,22]. The lack of league-level differences suggests that sleep problems are not restricted to elite sport but reflect broader structural and psychosocial demands present across competitive contexts [29].

A range of systemic factors may contribute to the high prevalence of poor sleep observed across all leagues. Training schedules in team sports often conflict with circadian preferences, especially when early morning training sessions or late competitions are required [43,44]. Environmental conditions common in competitive sport, including shared accommodation, noise, travel, and suboptimal sleeping conditions, further compromise sleep quality [17]. Psychological demands, such as pre-competition anxiety and the challenge of balancing athletic and non-sport roles, may additionally increase cognitive arousal and disrupt sleep [44]. These conditions are largely shared across competitive levels, which may explain the similar prevalence of sleep disturbances observed in all leagues. This finding underscores the importance of routine sleep screening to identify at-risk athletes and implement timely interventions, including sleep hygiene education, targeted behavioural strategies, and referrals to sleep specialists when necessary [45].

The main aim of the study was to examine whether sport experience, sleep quality, and coping strategies are associated with the level of perceived stress in the pre-competition period among female volleyball players. By analysing these interrelated variables simultaneously, the study provides a more comprehensive understanding of athletes’ psychological functioning during the pre-competition period and highlights the complex interplay among coping, sleep, and stress in female athletes. Regression analyses indicated that these variables collectively explained a meaningful proportion of stress variance. Less experienced athletes, those reporting poorer sleep quality, and those who more frequently sought social support experienced higher levels of perceived stress.

The negative association between sport experience and perceived stress suggests that accumulated experience provides psychological resources facilitating stress regulation. With increasing sport experience, coping strategies become more automated and less cognitively demanding, allowing athletes to manage competitive pressure more efficiently [6,7]. Experienced athletes are also more likely to rely on stable pre-competition routines and internalized regulatory strategies. In contrast, less experienced players may depend more strongly on interpersonal support as a compensatory mechanism when self-regulatory skills are still developing [35].

The positive association between poor sleep quality and elevated stress is consistent with the well-established bidirectional relationship between sleep and stress [20,46]. Sleep disturbances impair emotional regulation and cognitive functioning, which are essential for effective coping, while elevated stress increases physiological and cognitive arousal, further disrupting sleep [21,24]. This reciprocal process may create a self-reinforcing cycle in the pre-competition period. The finding that more frequent support-seeking was associated with higher perceived stress requires careful interpretation. Rather than indicating ineffective coping, this association may reflect the functional role of interpersonal coping in managing pre-competition tension. In team sport contexts, seeking support may primarily serve emotional regulation by helping athletes manage uncertainty, anticipatory worry, and heightened arousal, rather than directly reducing stress intensity [6,9]. Given the cross-sectional design, it is equally plausible that elevated perceived stress increases the likelihood of seeking support, rather than support-seeking contributing to higher stress levels [47]. Research indicates that team processes, coach–athlete relationships, and perceived social support can buffer against stress and burnout and enhance engagement [35]. Therefore, higher stress levels among athletes who more frequently seek support may reflect greater emotional engagement with the competitive situation rather than maladaptive coping [35,47]. In addition, the applied measure does not differentiate between proactive and reactive support-seeking, limiting conclusions as to whether support was mobilized in anticipation of stress or in response to its escalation [9,11]. Importantly, research in team sports suggests that support-seeking is a common and often adaptive response to shared competitive demands, embedded within collective coping processes and interpersonal regulation strategies [7,35,48]. The effectiveness of such strategies depends more on the quality, timing, and type of support than on its frequency alone [49]. For example, emotional support may help regulate pre-competition affect, whereas instrumental support may facilitate task-focused coping. Distinguishing between these forms of support represents an important direction for future research.

Taken together, the findings support an integrative perspective in which stress during the pre-competition period in female volleyball players emerges from the interaction of sport experience, sleep quality, and coping strategies rather than being explained solely by competitive level. Less experienced athletes may be particularly vulnerable due to less developed self-regulatory skills, while poor sleep further amplifies stress reactivity. Support-seeking plays a complex role that depends on context, timing, and quality, rather than on frequency.

Several limitations should be acknowledged. The cross-sectional design precludes conclusions about causal directionality among stress, sleep, and coping variables. Reliance on self-report measures increases susceptibility to response biases, although anonymity likely reduced social desirability effects. In addition, the lack of control for training load, match importance, travel, and menstrual cycle phase may have contributed to unexplained variance in stress and sleep outcomes [17,20]. Finally, although the PSS-4 and PSQI are well validated and widely used, they were not designed specifically for sport settings and may have limited sensitivity to competition-related stressors [34,50]. Future research should combine general and sport-specific measures and employ longitudinal or momentary designs to capture the dynamic nature of pre-competition stress more precisely.

## 5. Conclusions

This study adds to the literature on athlete mental health by indicating that less experienced female volleyball players tend to report higher levels of general perceived stress during the pre-competition period. Poorer sleep quality was also associated with higher perceived stress. These findings highlight the importance of addressing psychological and recovery-related factors when supporting athletes in competitive contexts, rather than focusing solely on competitive level.

The consistently high prevalence of poor sleep observed across all leagues suggests that sleep-related difficulties represent a systemic challenge in women’s volleyball. Monitoring sleep quality during the pre-competition period may provide useful information about athletes’ psychological functioning and well-being. Evidence from intervention studies indicates that improving sleep through behavioural and educational strategies may help reduce perceived stress and enhance subjective well-being, underscoring sleep as a modifiable target for athlete support programmes [41,51].

From an applied perspective, the findings emphasize the importance of supporting female athletes not only in physical and technical domains but also in psychological skills development. In particular, early psychological skills training may help less experienced athletes build effective coping resources and manage stress during competitive periods. Therefore, psychological skills training and sleep hygiene education represent important components of regular practice in female volleyball [19]. In practical terms, this may include coping-skills training, guided relaxation or imagery exercises, and regular sleep hygiene consultations embedded within the training process, while recognizing that sustained benefits are likely to require ongoing support and reinforcement.

Future research should extend these findings using longitudinal and intervention-based designs to clarify the temporal relationships between stress, sleep, and coping strategies. Incorporating sport-specific measures, objective sleep assessments (e.g., actigraphy), and momentary assessment tools may further enhance understanding of how athletes experience and regulate stress during the pre-competition period across different stages of athletic development.

## Figures and Tables

**Table 1:** Descriptive Characteristics of Female Volleyball Players Across Leagues.

	Tauron League		I League		II League	
Variable	M ± SD	Min–Max	M ± SD	Min–Max	M ± SD	Min–Max
Age	24.77 ± 5.08	18–35	23.07 ± 3.51	18–30	21.2 ± 3.66	18–29
Sport experience	13.19 ± 5.26	6–25	1.17 ± 0.59	5–20	10.2 ± 3.75	3–19
Perceived stress	6.03 ± 2.27	2–10	6.67 ± 2.34	0–12	6.77 ± 2.50	1–12
Sleep quality (PSQI Score)	5.00 ± 2.28	1–10	5.33 ± 1.97	3–11	6.33 ± 2.99	1–12
Subjective sleep quality	0.97 ± 0.66	0–3	1.10 ± 0.55	0–2	1.17 ± 0.59	0–2
Sleep latency	1.16 ± 0.86	0–3	1.17 ± 0.95	0–3	1.10 ± 0.96	0–3
Sleep duration	0.10 ± 0.30	0–1	0.10 ± 0.31	0–1	0.67 ± 0.80	0–3
Sleep efficiency	0.29 ± 0.69	0–3	0.70 ± 1.12	0–3	0.70 ± 0.92	0–3
Sleep disturbances	1.00 ± 0.45	0–2	1.13 ± 0.51	0–3	1.17 ± 0.65	0–3
Use of sleep medication	0.13 ± 0.56	0–3	0.10 ± 0.40	0–2	0.30 ± 0.92	0–3
Daytime dysfunction	1.35 ± 0.71	0–3	1.03 ± 0.81	0–3	1.23 ± 0.90	0–3
Setting the goal/victory	3.95 ± 0.59	2.20–5.00	3.66 ± 0.92	1.60–5.00	3.77 ± 0.95	1.00–5.00
Seeking support	2.90 ± 0.95	1.00–4.71	3.02 ± 0.85	1.14–4.71	2.66 ± 1.06	1.00–5.00
Applying mental techniques	3.13 ± 0.91	1.25–4.75	2.76 ± 1.10	1.00–4.75	2.37 ± 1.08	1.00–4.50
Planning/focus on activity	3.66 ± 0.59	2.00–4.50	3.38 ± 0.82	1.17–4.67	3.26 ± 0.99	1.00–4.83

**Table 2:** Pearson R correlation matrix for the continuous variables.

	Sport Experience	Perceived Stress	Sleep Quality	Setting the Goal/Victory	Seeking Support	Applying Mental Techniques
Perceived stress	−0.26 *	-				
Sleep quality	−0.21 *	0.29 *	-			
Setting the goal/victory	−0.21 *	−0.11	0.01	-		
Seeking support	0.10	0.09	−0.07	0.27 *	-	
Applying mental techniques	0.15	−0.16	−0.04	0.23 *	0.31 **	-
Planning/focus on activity	0.00	−0.04	−0.01	0.57 ***	0.60 ***	0.48 ***

Notes: Statistically significant associations: * *p* = 0.05, ** *p* = 0.005, *** *p* < 0.001.

**Table 3:** Results of the regression analysis predicting the level of perceived stress by sleep quality, coping strategies, and sports experience.

Variable	β	SE ^1^	t (85)	*p*
Sleep quality	0.25	0.10	2.50	0.015
Sport experience	−0.25	0.10	−2.40	0.019
Setting the goal/victory	−0.19	0.11	−1.82	0.072
Seeking support	0.23	0.11	2.21	0.030
Applying mental techniques	−0.14	0.10	−1.36	0.178

^1^ SE—standard error.

## Data Availability

The data presented in this study are available in the RepOD repository, Version 1, at https://doi.org/10.18150/DOXUYD.

## Abbreviations

The following abbreviations are used in this manuscript:

SR3S	Stress Coping Strategies in Sport Questionnaire
PSS-4	Perceived Stress Scale
PSQI	Pittsburgh Sleep Quality Index
